# Selected *ABCB1*, *ABCB4* and *ABCC2* Polymorphisms Do Not Enhance the Risk of Drug-Induced Hepatotoxicity in a Spanish Cohort

**DOI:** 10.1371/journal.pone.0094675

**Published:** 2014-04-14

**Authors:** Eugenia Ulzurrun, Camilla Stephens, Francisco Ruiz-Cabello, Mercedes Robles-Diaz, Pablo Saenz-López, Hacibe Hallal, German Soriano, Eva Roman, M. Carmen Fernandez, M. Isabel Lucena, Raúl J. Andrade

**Affiliations:** 1 S Farmacología Clínica and UGC de Gastroenterología y Hepatología, Instituto de Investigación Biomédica de Málaga (IBIMA), Hospital Universitario Virgen de la Victoria, Universidad de Málaga, Málaga, Spain; 2 Centro de Investigación Biomédica en Red de Enfermedades Hepáticas y Digestivas (CIBERehd), Barcelona, Spain; 3 Departamento de Bioquímica y Biología Molecular III/Inmunología, Hospital Universitario Virgen de las Nieves, Universidad de Granada, Granada, Spain; 4 Instituto de Investigación Biosanitario de Granada, Granada, Spain; 5 Servicio de Aparato Digestivo, Hospital Morales Meseguer, Murcia, Spain; 6 Servicio de Gastroenterología, Hospital de la Santa Creu i Sant Pau, Universitat Autònoma de Barcelona, Barcelona, Spain; 7 Escola Universitària d'Infermeria EUI-Sant Pau, Universitat Autònoma de Barcelona, Barcelona, Spain; 8 Unidad de Gestión Clínica de Farmacia, Hospital Torrecárdenas, Almería, Spain; CIMA. University of Navarra, Spain

## Abstract

**Background and Aims:**

Flawed ABC transporter functions may contribute to increased risk of drug-induced liver injury (DILI). We aimed to analyse the influence of genetic variations in ABC transporters on the risk of DILI development and clinical presentations in a large Spanish DILI cohort.

**Methods:**

A total of ten polymorphisms in *ABCB1* (1236T>C, 2677G>T,A, 3435T>C), *ABCB4* (1954A>G) and *ABCC2* (−1774G>del, −1549A>G, −24C>T, 1249G>A, 3972C>T and 4544G>A) were genotyped using Taqman 5′ allelic discrimination assays or sequencing in 141 Spanish DILI patients and 161 controls. The influence of specific genotypes, alleles and haplotypes on the risk of DILI development and clinical presentations was analysed.

**Results:**

None of the individual polymorphisms or haplotypes was found to be associated with DILI development. Carriers homozygous for the *ABCC2* −1774del allele were however only found in DILI patients. Hence, this genotype could potentially be associated with increased risk, though its low frequency in our Spanish cohort prevented a final conclusion. Furthermore, carriers homozygous for the *ABCC2* −1774G/−1549A/−24T/1249G/3972T/4544G haplotype were found to have a higher propensity for total bilirubin elevations when developing DILI.

**Conclusions:**

Our findings do not support a role for the analysed polymorphisms in the *ABCB1*, *ABCB4* and *ABCC2* transporter genes in DILI development in Spanish patients. The *ABCC2* −1774deldel genotype was however restricted to DILI cases and could potentially contribute to enhanced DILI susceptibility.

## Introduction

Drug-induced liver injury (DILI) is an adverse drug reaction of special concern due to its potentially severe consequences. Despite increased research in this area over the last decades, the underlying mechanisms of DILI are not fully understood. However, host genetic variations are believed to be essential components in DILI development. Many DILI studies to date have focused on variations in genes involved in phase I (bioactivation) and II (detoxification) of drug metabolism and several potential risk alleles have been identified [1]. Increasing attention is being paid to the role of phase III (elimination) proteins in DILI. This group consists mainly of ATP-binding cassette (ABC) transporters, which are involved in the cellular efflux of various endogenous and exogenous compounds, including therapeutic drugs. The number of transporters in the cell membrane and their functional level can therefore potentially influence drug pharmacokintetics/pharmacodynamics and lead to interindividual differences in drug elimination.

Among the transporters the most investigated for its relationship with DILI is the bile salt export pump protein (BSEP), which mediates the efflux o bile acids from hepatocytes into the bile canaliculus. Interference with BSEP function using *in vitro* models has been shown to be highly predictive of liver injury in drug development [2]. Besides, a genetic variation in *ABCB11*, which encodes BSEP, has been linked to the risk of hepatocellular DILI in a large population of DILI patients from a Spanish Registry [3].

Less is known regarding the role of other transporters in hepatotoxicity. P-glycoprotein (P-gp) is the major drug transporter recognizing a wide range of substrates. In the liver P-gp is located in the canalicular membrane, where it facilitates biliary drug excretion. This transporter is encoded by the *ABCB1* gene, which contains 29 exons in a genomic region spanning 209.6 kb [4]. P-glycoprotein is a well-known determinant in multidrug resistance, in particularly in chemotherapy. Overexpression in tumour cells can lead to enhanced drug excretion, which subsequently inhibits the drug to reach a pharmacologically active concentration [5]. In addition, *ABCB1* has been suggested to play a role in specific forms of hepatotoxicity [6]–[8].

The multidrug resistance protein 2 (MRP2), encoded by the *ABCC2* gene, is likewise involved in drug transport. It is confined to the apical membrane of various polarized cells and subsequently involved in the final stage of cellular detoxification. Similar to P-gp, this ABC transporter has broad substrate specificity, comprising of many conjugated organic anions and drug metabolites. In addition, MRP2 also plays an essential part in bilirubin glucuronoside and bile salt sulfate/glucuronoside biliary excretion [9], [10]. The role of *ABCC2* polymorphisms in DILI is currently unclear with controversial results between studies [11], [12].

Multidrug resistance protein 3 (MDR3) is encoded by the *ABCB4* gene and highly expressed in the liver. This ABC transporter is not believed to play a major role in drug transport, but is essential for biliary phosphatidylcholine (PC) secretion. PC combines with bile salts to form mixed micelles, which prevent against damage caused by detergent-like bile salts activity. With insufficient PC concentration, bile salts transported into the bile by BSEP can potentially solubilize cell membranes resulting in biliary toxicity [13]. Hence, genetic variations comprising MRD3 activity could potentially lead to DILI, as demonstrated for BSEP.

Due to the participation of ABC transporters in drug elimination and their known capacity to produce serious hepatic conditions [14], [15], flawed ABC transporter functions as a consequence of genetic variations could contribute to an increased risk of drug-induced hepatotoxicity. In this study we have focused on ten polymorphisms located in *ABCB1*, *ABCB4* and *ABCC2*. The *ABCC2* polymorphisms were selected based on previous DILI association studies in ethnically different cohorts [11], [12]. The functional effects of the polymorphisms in the other two genes are relatively unknown with regards to DILI. However, previous studies have shown that the selected *ABCB1* polymorphisms may affect serum drug concentration [16], [17]. In addition, the selected *ABCB4* polymorphism changes an evolutionary conserved amino acid, which could lead to altered protein physiochemical properties [18]. Hence, our aim in this study was to analyse the influence of selected genetic variations in ABC transporters on the risk of DILI development and clinical presentations in a large Spanish DILI cohort.

## Material and Methods

### Subjects and study protocol

A total of 141 DILI cases were selected from those submitted to the Spanish DILI Registry, a collaborative network established in 1994 to prospectively identify cases of DILI in a standardized manner. The criteria for DILI at the time of subject inclusions in the study were: an increase in alanine transaminase (ALT) ≥3 times the upper limit of normal (xULN) or ≥2 xULN of alkaline phosphatase (ALP). However, 93% of the cases also fulfilled the more recent DILI criteria established by Aithal and coworkers: ALT ≥5 xULN or ALT ≥3 xULN + total bilirubin (TBL) ≥2 xULN or ALP ≥2 xULN [19]. The pattern of liver injury was classified based on R value calculations as previously described [20]. A detailed description of the operational structure of the registry, data recording and case ascertainment has been reported elsewhere [21]. As a control group we selected a total of 161 healthy Spanish subjects, unrelated to the DILI patients, without previous DILI history. These controls were recruited from blood donors in the Spanish Bone Marrow Donor Registry from the same geographic region and of similar age and gender distribution.

### Ethics statement

The study protocol was approved by the local ethics committee of the coordinating center at the Hospital Universitario Virgen de la Victoria (Comité Ético de Investigación) in Málaga, Spain. All subjects who took part in the study were over 18 years of age. All subjects gave written informed consent, which conformed to the current Helsinki Declaration, prior to study enrolment.

### DNA extraction and genotyping

Venous blood was obtained from each subject and DNA was extracted as described previously [22]. Samples and controls were genotyped for the *ABCB1* c.1236T>C (p.Gly412Gly, rs1128503), c.3435T>C (p.Ile1145Ile, rs1045642), *ABCB4* c.1954A>G (p.Arg652Gly, rs2230028) and *ABCC2* c.-1549A>G (rs1885301), c.-24C>T (rs717620), c.1249G>A (p.Val417Ile, rs2273697), c.3972C>T (p.Ile1324Ile, rs3740066) and c.4544G>A (p.Cys1515Tyr, rs8187710) using a validated 5′-nuclease PCR based assay with allele specific fluorescent probes (TaqMan SNP Genotyping Assays, Applied Biosystems, Foster City, CA, USA) as previously described [23]. In short, 10 ng of sample DNA in 25 µL of reaction solution containing 12.5 µL of the 2× Taqman Universal PCR Mix (Applied Biosystems), and 1.25 µL of pre-developed assay reagent from the SNP genotyping product (Applied Biosystem), containing two primers and two MGB-Taqman probes was used for each sample reaction. Reaction conditions consisted of pre-incubation at 95°C for 10 min followed by 40 cycles of 95°C for 15 s and 60°C for 1 min.

Due to the nature of the *ABCB1* 2677G>T/A (p.Ala893Ser/Thr, rs2032582) and *ABCC2* -1774G>del (rs369192412) polymorphisms, these were genotyped by direct sequencing with Big dye terminator cycle sequencing kit (Applied Biosystems, Foster City, CA, USA) of a 224 and 554 basepair amplicon, respectively, encompassing the polymorphism of interest. The primers used for sequencing were 5′-AGGTTCCAGGCTTGTTGTAATTAC-3′/5′-GGAAGAACAGTGTGAAGACAATGG-3′ (rs2032582) and 5′-GAAAATGATGGAGAAACTGCCATA-3′/5′-ATGACAACTGAATCAACTTCACAA-3′ (rs369192412).

### Statistical analysis

Genotype distributions and allelic frequencies were analysed in DILI patients and controls using the comparison of proportions test, a derivative of the Fisher's exact test. Bonferroni's correction for multiple tests, whereby the probability value (*P*) was multiplied by the number of genotypes or alleles compared to give a corrected *P* value (*Pc*), was performed in order to account for problems of significant associations arising by chance after multiple comparisons. Haplotype frequencies were performed for the *ABCC2* and *ABCB1* polymorphisms using the Haploview 4.1 software (Broad Institute, Cambridge, MA, USA). Due to software incompatibility with three allelic polymorphisms the ABCB1 2677G>T,A was considered as 2677G>T. Hence, six cases and seven controls with an *ABCB1* 2677A allele were omitted from the haplotype analysis. Allele combination frequencies formed by *ABCC2*/*ABCB1* and *ABCB1*/*ABCB4* in 97 DILI patients and 125 controls were also predicted using Haploview 4.1.

Means were compared by Student's t test for independent sample. Analysis of variance (ANOVA) was used for comparison of groups. Where variables did not follow a normal distribution, a nonparametric Kruskal-Wallis analysis was performed. All analyses were performed using the SPSS 19.0 statistical software package (SPSS Inc, Chicago, IL, USA) and *p*<0.05 was considered to be statistically significant. Statistical power calculations were performed using the Episheet program (krothman.hostbyet2.com/epishhet.xl).

## Results

### DILI patient characteristics

A total of 141 DILI patients were included in the study of which 73 were males and 68 females. The mean age at DILI onset was 54 years, ranging from 14 to 83 years. The predominant pattern of injury, based on the R value calculated from the first blood sample analysis after DILI onset, was hepatocellular (n = 77) followed by mixed (n = 36) and cholestatic (n = 28). The main causative therapeutic drug group was antiinfectives (37%), followed by musculo-skeletal (13%), nervous system (12%), cardiovascular (11%) and antineoplastic drugs (7%). Of the 141 patients only one developed fulminant hepatic failure and required a liver transplantation. Applying the Council for International Organizations of Medical Science (CIOMS) causality scale the 141 cases were classified as 78 (55%) highly probable, 56 (40%) probable and 7 (5%) possible DILI cases. Demographics, clinical and laboratory parameters of the 141 DILI cases included in the study are outlined in Table 1.

**Table 1:** Demographics, clinical and laboratory parameters of the 141 DILI cases included in the study.

Demographics	
Mean age, years (range)	54 (14–83)
Male/female	73/68
Body mass index, mean ±SD	26.7±4.3
***Time to DILI onset***	
< 30 days	77 (54.6%)
30–90 days	36 (25.5%)
>90 days	28 (19.9%)
***Clinical presentation, *** **n (%)**	
Jaundice	97 (68.8%)
Hospital admission	75 (53.2%)
***Type of liver injury, *** **n (%)**	
Hepatocellular	81 (57.4%)
Cholestatic	27 (19.2%)
Mixed	33 (23.4%)
Biochemical parameters*, **mean ±SD (median)**	
TBL xULN	7.0±7.7 (4.3)
ALT xULN	18.5±21.5 (9.1)
ALP xULN	2.4±2.0 (1.7)

*First available serum analysis after DILI onset, TBL: total bilirubin, ALT: alanine transaminase, ALP: alkaline phosphatase, xULN: times the upper limit of normal

### *ABCB1, ABCB4* and *ABCC2* polymorphisms

In search for genetic differences DILI patients and controls were genotyped for 10 specific polymorphisms located in *ABCB1* (1236T>C, 2677G>T/A and 3435T>C), *ABCB4* (1954A>G) and *ABCC2* (-1774G>del, -1549A>G, -24C>T, 1249G>A, 3972C>T and 4544G>A). No significant differences were found in either genotype distribution or allele frequency between cases and controls for any of the individual polymorphisms (Table 2). The statistical power to find significant differences with an odds ratio of 2 in our cohort was 78%–88%, except for the less polymorphic *ABCB1* 2677G>A (24%), *ABCB4* 1954A>G (65%) and *ABCC2* -1774G>del (65%) and 4544G>A (59%). The *ABCC2* -1774deldel genotype was not present in any of the controls, which prevented a statistical analysis using the comparison of proportions method. However, when analysing the *ABCC2* -1774deldel distribution between cases and controls using the Fisher's exact test the difference was found to be statistically significant (*p* = 0.014). As the MRP2 transporter, encoded by *ABCC2*, is known to transport various conjugated drug metabolites a smaller subgroup of 22 DILI cases induced by drugs undergoing glucuronidation was also analysed. None of the alleles corresponding to the six *ABCC2* polymorphisms were however found to correlate with DILI risk in this subgroup (data not shown).

**Table 2:** Genotype distribution and allele frequency of 10 polymorphisms situated in the genes *ABCC2* (-1771G>delG, -1549G>A, -24C>T, 1249G>A, 3972C>T, 4544G>A), *ABCB1* (1236T>C, 2677G>T/A, 3435T>C) and *ABCB4* (1954A>G).

Genotype distribution, n (%)				Allele frequency, n (%)		
Polymorphism		DILI	Control		DILI	Control
***ABCC2***		n = 117	n = 156		n = 234	n = 312
*c.-1774G>del*						
	GG	100 (85)	123 (79)	G	212 (91)	279 (89)
	Gdel	12 (10)	33 (21)	delG	22 (9)	33 (11)
	deldel	5 (4)	0 (0)			
*c.-1549G>A*						
	GG	34 (29)	36 (23)	G	115 (49)	144 (46)
	GA	47 (40)	72 (46)	A	119 (51)	168 (54)
	AA	36 (31)	48 (31)			
*c.-24C>T*						
	CC	66 (56)	86 (55)	C	172 (73)	229 (73)
	CT	40 (34)	57 (37)	T	62 (26)	83 (27)
	TT	11(9)	13 (8)			
*c.1249G>A*						
	GG	73 (62)	106 (68)	G	183 (78)	257 (82)
	GA	37 (32)	45 (29)	A	51 (22)	55 (18)
	AA	7 (6)	5 (3)			
*c.3972C>T*						
	CC	34 (29)	48 (31)	C	123 (53)	168 (54)
	CT	55 (47)	72 (46)	T	111 (47)	144 (46)
	TT	28 (24)	36 (23)			
*c.4544G>A*						
	GG	104 (89)	130 (83)	G	220 (94)	284 (91)
	GA	12 (10)	24 (15)	A	14 (6)	28 (9)
	AA	1 (1)	2 (1)			
***ABCB1***		n = 119	n = 152		n = 238	n = 304
*c.1236T>C*						
	TT	17 (14)	28 (18)	T	88 (37)	119 (39)
	CT	54 (45)	63 (41)	C	150 (63)	185 (61)
	CC	48 (40)	61 (40)			
*c.2677G>T, A*						
	GG	44 (37)	64 (42)	G	146 (61)	190 (62)
	GT	50 (42)	58 (38)	T	83 (35)	107 (35)
	GA	8 (7)	4 (3)	A	9 (4)	7 (2)
	TT	16 (13)	23 (15)			
	TA	1 (1)	3 (2)			
	AA	0 (0)	0 (0)			
*c.3435T>C*						
	TT	19 (16)	29 (19)	T	95 (40)	126 (41)
	CT	57 (48)	68 (45)	C	143 (60)	178 (59)
	CC	43 (36)	55 (36)			
***ABCB4***		n = 134	n = 161		n = 268	n = 322
*c.1954A>G*						
	AA	112 (84)	131 (81)	A	246 (92)	290 (90)
	AG	22 (16)	28 (17)	G	22 (8)	32 (10)
	GG	0 (0)	2 (1)			

### Linkage disequilibrium (LD) and haplotype analysis

**Figure 1:**
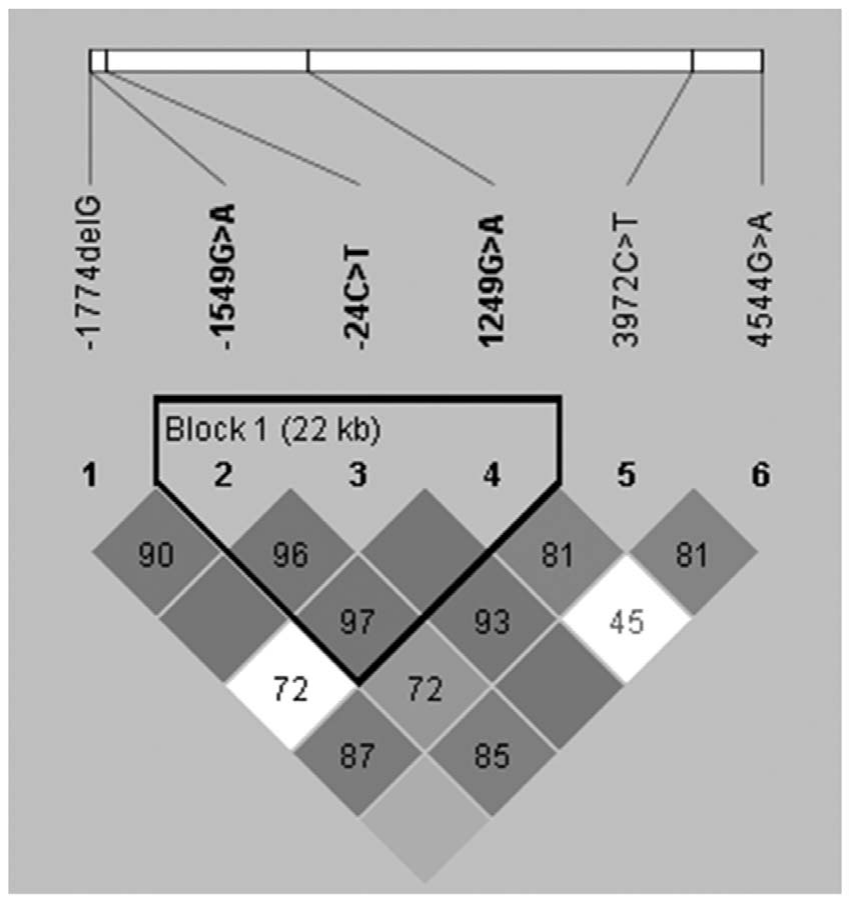
Linkage disequilibrium (LD) plot of *ABCC2* polymorphisms. Each square represents a pairwise LD relationship formed between the two single polymorphisms with D' values multiplied by 100 shown inside the squares. The shade of grey indicates the degree of LD significance. Dark grey squares represent statistically significant LD as measured by D' up to a maximum of 1. White squares indicate D' values less than 1 with no statistically significant LD. The three polymorphisms -1549G>A, -24C>T and 1249G>A were found to form a separate haploblock with evidence of strong LD according to the Gabriel algorithm.

**Figure 2:**
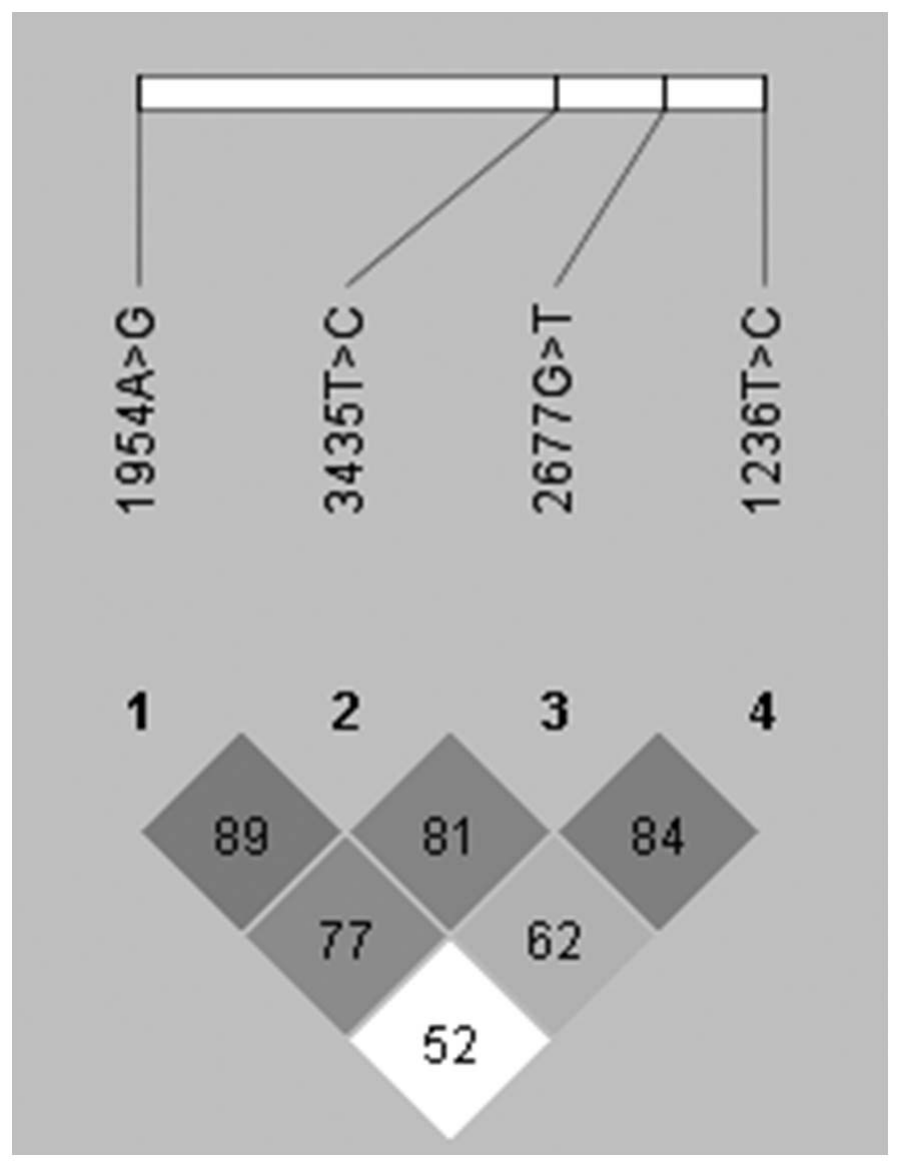
Linkage disequilibrium (LD) plot of *ABCB1* (1236T>C, 2677G>T, 3435T>C) and *ABCB4* (1954A>G) polymorphisms. Each square represents a pairwise LD relationship formed between the two single polymorphisms with D' values multiplied by 100 shown inside the squares. The shade of grey indicates the degree of LD significance. Dark grey squares represent statistically significant LD as measured by D' up to a maximum of 1. White squares indicate D' values less than 1 with no statistically significant LD.

Strong pairwise LD was seen between several of the *ABCC2* polymorphisms in particular among -1549A>G, -24C>T and 1249G>A, which formed a haplotype block (Figure 1). The major haplotypes formed by the six *ABCC2* polymorphisms were equally distributed between DILI patients and controls (Table 3). Similar results were obtained when analysing haplotype frequencies formed by the three SNPs constituting the haploblock (data not shown). The three *ABCB1* SNPs were found to be in LD, with D' values of 0.84, 0.81 and 0.62 for the SNP pairs 1236T>C/2677G>T, 2677G>T/3435T>C and 1236T>C/3435T>C, respectively (Figure 2). The *ABCB4* 1954A>G polymorphism was also included in the LD analysis due to *ABCB4* being adjacent to *ABCB1* on chromosome 7. The *ABCB4* 1954A>G and *ABCB1* 3435 T>C SNP pair demonstrated a D' value of 0.89, thought the R^2^ value only reached 0.058 (Figure 2).

**Table 3:** Predicted haplotype frequencies of transporter gene polymorphisms.

-1774G>del	-1549A>G	-24C>T	1249G>A	3972C>T	4544G>A	DILI (2n = 234)	Controls (2n = 312)	*p*
G	A	**T**	G	T	G	58 (25%)	78 (25%)	0.944
G	**G**	C	**A**	C	G	42 (18%)	48 (15%)	0.444
G	**G**	C	G	C	G	36 (15%)	46 (15%)	0.805
G	A	C	G	**T**	G	34 (14%)	44 (14%)	0.927
**del**	**G**	C	G	C	G	18 (7.7%)	29 (9.3%)	0.548
G	A	C	G	C	**A**	13 (5.6%)	21 (6.7%)	0.571

Major haplotypes formed by *ABCC2* polymorphisms -1774G>del, -1549A>G, -24C>T, 1249G>A, 3972C>T and 4544G>A in DILI patients and controls.

The table outlines major haplotypes with a predicted frequency higher than 5% in the control population. The minor alleles in the study cohort are marked in underlined bold faced letters.

Similar to the results for the *ABCC2* haplotypes, none of the major *ABCB1* haplotypes differed between DILI patient and controls in terms of frequency (Table 4). Allele combinations formed by the studied *ABCC2* and *ABCB1* polymorphisms as well as *ABCB1* and *ABCB4* were also analysed in search for distinguishing genetic features. No allele combinations formed by the different transporter genes presented any significant associations with DILI development (Table 5 and Table 6).

**Table 4:** Predicted haplotype frequencies of transporter gene polymorphisms.

1236T>C	2677G>T	3435T>C	DILI (2n = 220)	Controls (2n = 290)	*p*
C	G	C	114 (52%)	138 (48%)	0.343
**T**	**T**	**T**	69 (31%)	82 (28%)	0.475
**T**	G	C	15 (6.8%)	24 (8.3%)	0.563
C	G	**T**	7 (3.2%)	18 (6.2%)	0.123

Major haplotypes formed by *ABCB1* polymorphisms 1236C>T, 2677G>T and 3435T>C in DILI patients and controls.

The table outlines major haplotypes with a predicted frequency higher than 5% in the control population.

The minor alleles in the study cohort are marked in underlined bold faced letters.

**Table 5:** Predicted allele combination frequencies of transporter gene polymorphisms.

ABCC2						ABCB1			DILI (2n = 194)	Controls (2n = 250)	P
-1774G>del	-1549A>G	-24C>T	1249G>A	3972C>T	4544G>A	1236C>T	2677G>T	3435C>T			
G	A	**T**	G	**T**	G	C	G	C	27 (14%)	26 (10%)	0.213
G	A	**T**	G	**T**	G	**T**	**T**	**T**	15 (7.7%)	23 (9.2%)	0.647
G	A	C	G	**T**	G	C	G	C	16 (8.2%)	20 (8.0%)	0.9.25
G	**G**	C	**A**	C	G	C	G	C	15 (7.7%)	19 (7.6%)	0.9.30
G	**G**	C	G	C	G	C	G	C	15 (7.7%)	17 (6.8%)	0.716
G	A	C	G	**T**	G	**T**	**T**	**T**	12 (6.2%)	16 (6.4%)	0.949
G	**G**	C	**A**	C	G	**T**	**T**	**T**	13 (6.7%)	13 (5.2%)	0.513
**del**	**G**	C	G	C	G	C	G	C	9 (4.6%)	13 (5.2%)	0.799

Major allele combination formed by *ABCC2* (-1774G>del, -1549A>G, -24C>T, 1249G>A, 3972C>T and 4544G>A) and *ABCB1* (1236C>T, 2677G>T and 3435T>C) polymorphisms in DILI patients and controls.

The table outlines major allele combinations with a predicted frequency higher than 5% in the control population. The minor allele in the study cohort is marked in underlined bold faced letters.

**Table 6:** Predicted allele combination frequencies of transporter gene polymorphisms.

ABCB1			ABCB4	DILI (2n = 194)	Controls (2n = 250)	P
1236C>T	2677G>T	3435C>T	1954A>G			
C	G	C	A	88 (45%)	96 (38%)	0.141
**T**	**T**	**T**	A	60 (31%)	78 (31%)	0.991
C	G	C	**G**	12 (6.2%)	20 (8.0%)	0.456
**T**	G	C	A	13 (6.7%)	17 (6.8%)	0.963
C	G	**T**	A	6 (3.1%)	14 (5.6%)	0.197

Major allele combination formed by *ABCB1* (1236C>T, 2677G>T and 3435T>C) and *ABCB4* 1954A>G polymorphisms in DILI patients and controls.

The table outlines major allele combinations with a predicted frequency higher than 5% in the control population. The minor allele in the study cohort is marked in underlined bold faced letters.

### ABC transporter gene polymorphisms and clinical parameters

**Figure 3:**
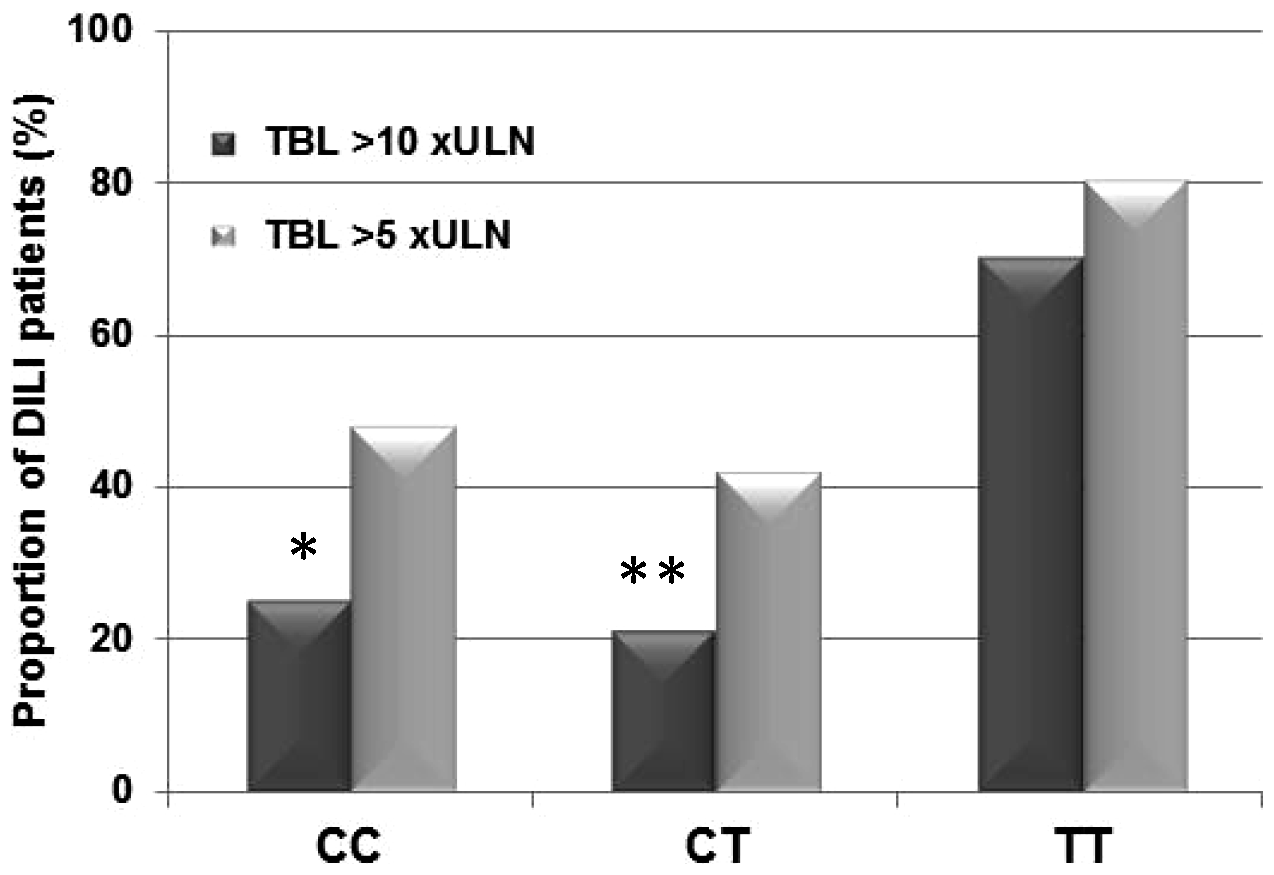
Serum total bilirubin elevations in 110 Spanish DILI patients classified by *ABCC2* -24C>T genotypes. Bilirubin measurements from the first available blood test after DILI initiation was used for this analysis. Footnote: *p = 0.0074 (CC vs TT), **p = 0.0059 (CT vs TT), TBL: total bilirubin, xULN: times the upper limit of normal.

Phenotypic parameters, such as type of liver injury, serum total bilirubin (TBL), alanine transaminase (ALT) and alkaline phosphatase (ALP) values, at presentation were analysed according to genotypes in the 10 different polymorphisms. Patients with the *ABCC2* -24TT genotype (n = 10, 1 case without TBL data) were found to have a higher tendency towards elevated TBL values with a mean value of 12.2 xULN vs 6.5 (CC, n = 63, 3 cases without TBL data) and 6.1 (CT, n = 37, 3 cases without TBL data) (*p* = 0.06) at presentation. The -24TT carriers also demonstrated higher maximum TBL, although not reaching statistical significance (TT: 12.5 vs CC: 7.2 and CT: 7.1 xULN) (*p* = 0.23). Seventy percent of the TT carriers reached a maximum TBL value of more than 10 xULN, while the corresponding proportion for the CC and CT carriers were 25% and 21%, respectively (*p* = 0.011) (Figure 3). Post hoc analyses with the Fisher's exact test demonstrated statistically significant differences between CC and TT carriers (*p* = 0.0074) as well as CT and TT carriers (*p* = 0.0059) in terms of proportion of cases with bilirubin levels above 10 xULN. In addition to being more prone to higher TBL elevations, all the *ABCC2* -24TT carriers shared the same *ABCC2* diplotype, being homozygous for the -1774G, -1549A, -24T, 1249G, 3972T and 4544G alleles and 73% developed hepatocellular type of injury.

Patients homozygous for the 1249A allele (n = 7) were all found to have developed hepatocellular injury, but only 29% reached a maximum TBL value of more than 5 xULN. All of these patients were also homozygous for the -1549G and -24C alleles with the majority being homozygous for the -1774G, 3972C and 4544G alleles. No further clinical or biochemical parameters were found to differ between genotypes and alleles for any of the analysed *ABCB1*, *ABCB4* and *ABCC2* polymorphisms.

## Discussion

Based on their crucial role in drug metabolism and maintenance of liver homeostasis ABC transporters could play a role in DILI development. A defect in any of these transporters can affect biliary clearance and subsequently delay the elimination of toxic substances, including drug metabolites. This has been confirmed through diseases such as intrahepatic cholestasis and Dubin-Johnson syndrome caused by mutations in *ABCB4* and *ABCC2*, respectively. In this study we analysed a total of 10 polymorphisms located in *ABCB1*, *ABCB4* and *ABCC2* in DILI patients and healthy controls and found that none of the individual polymorphisms were associated with DILI development. The statistical power to detect differences with an odds ratio of 2 was relatively high (>75%) for most of the polymorphisms. However, in the polymorphisms with lower minor allele frequencies and consequently lower statistical power, in particular *ABCB1* 2677G>A and *ABCC2* 4544G>A, it is possible that this may have contributed to the inability to detect significant differences between the cases and controls. Hence, to ascertain that these polymorphisms have no role in DILI development, despite our findings, requires larger cohorts. Furthermore, our controls were not drug-matched and although healthy at the time of the blood extraction (and having no recorded DILI episode) the possibility that some of them could develop DILI at a later stage cannot be absolutely discarded. Due to the low incidence rate of idiosyncratic DILI (1/10 000–1/100 000 user of a specific treatment) however, we hold it for very unlikely that a large proportion of the controls would be susceptible to developing DILI. In addition, the main causative agents in our DILI cases were antiinfectives and musculo-skeletal drugs, which are widely used in the Spanish community. Hence, it is likely that many of the controls in fact have been exposed to the causative agents corresponding to the DILI cases. In an earlier study we compared genotype data of an additional ABC transporter polymorphism in this control cohort with those of controls with recorded drug intakes and found no significant differences between the two cohorts [3].

We found, however, that there was a tendency towards a DILI association for homozygous *ABCC2* -1774del carriers as this genotype was not present in any of the controls. Evidently, this needs to be confirmed in a larger cohort, which could be a difficult task due to the rarity of this genotype in conjunction with DILI itself being a relatively rare condition. Nevertheless, the -1774deldel genotype has been found to play a role in cholestatic/mixed type of DILI development in a Korean population, where this genotype is more common than seen in Caucasians [11]. The same study also demonstrated that the -1774del allele reduced the level of gene expression compared to the G allele. Furthermore, the -1774G>del polymorphism has been shown to be in strong linkage disequilibrium with rs3740065, a polymorphism located in intron 29 of the same gene, in a Japanese population [24]. Interestingly, the rs3740065 polymorphism provided the highest association among genes involved in drug metabolism in a recent genome-wide association study on 783 DILI patients [25]. Although, the extent of linkage disequilibrium between -1774G>del and rs3740065 in Caucasians is currently unknown.

Koreans homozygous for the *ABCC2* -1549A and 3972T alleles appear to be more prone to developing hepatocellular type of DILI [11], though no associations were seen with these genotypes in the Spanish cohort. Substantial differences in allele frequencies between the Korean and our control cohort may have contributed to the discrepant results. In particular, 3972C>T has been shown to vary considerably between Asians and Caucasians with regards to allele and associated haplotype frequencies [24]. It is also possible that DILI risk alleles differ depending on the causal agent. The Korean cohort, in which various *ABCC2* risk alleles were identified, contained mostly herbal remedy-induced hepatotoxicity cases, while our cohort included a larger variety of causal agents, mainly conventional pharmaceuticals. The presence of drug-specific risk alleles is supported by a recent analysis of seven *ABCC2* polymorphisms in a different Korean cohort consisting of antituberculosis treatment-induced hepatotoxicity cases. This study could not reproduce the results found in the earlier Korean cohort, as no associations were found for any of the individual polymorphisms or haplotypes [26]. Our results also differ from those of Daly and coworkers, who found a significant variation in the *ABCC2* -24C>T genotype distribution between British DILI patients and controls [12]. The fact that the British study was restricted to diclofenac-induced DILI and only 3 of our 117 cases analysed were due to diclofenac may have attributed to the different findings.

No *ABCC2* haplotypes were found to be over- or underrepresented in the DILI cohort. However, a smaller group of patients with substantial TBL elevations were identified based on sharing an identical diplotype (-1774GG/-1549AA/-24TT/1249GG/3972TT/4581GG). The *ABCC2* -24T/1249G/3972T haplotype has been shown to yield lower protein expression and efflux rate *in vitro* compared to the more common -24C/1249G/3972C haplotype, presumably due to variations in mRNA secondary structures and resultant posttranscriptional modifications [27]. One might speculate that the extended *ABCC2* haplotype -1774G/-1549A/-24T/1249G/3972T/4544G has a similar functional effect, based on indications of reduced expression for the -1549A and 4544G allele [11], [28]. This haplotype, however, does not appear to enhance the risk of DILI development as it was seen to be the most common haplotype present in both cases and controls. Neither was the homozygous diplotype more prevalent in the DILI cases. Nevertheless, the higher TBL values seen with this diplotype could be an effect of reduced ABCC2 activity, due to having two copies of a haplotype assumed to decrease the transporter function, in addition to the cellular strain of DILI. The formation of reactive metabolites from the parent drug is combated through detoxification processes, which often involve glutathione conjugation [29]. Such glutathione conjugates could then compete with conjugated bilirubin for MRP2 biliary excretion, leading to bilirubin being released back into the blood stream by basolateral transporters, such as MRP3 encoded by the gene *ABCC3*, and consequently elevating serum concentrations of bilirubin glucuronosides. Conjugated bilirubin transport by MRP3 has been demonstrated and hepatic MPR3 induction has been found in situations of decreased MRP2 activity [30], [31].

Allele distributions of the *ABCB1* polymorphisms focused on in this study are known to vary considerably between different ethnic populations. However, our results were comparable to those established earlier in Caucasian populations [32]. The functional effects of these polymorphisms and their roles in drug transport are still controversial [33]. Nevertheless, the 3435T allele is thought to cause translational problems due to the formation of a rare codon, affecting the timing of cotranslational folding and membrane insertion and consequently could affect substrate binding and specificity, despite being a synonymous polymorphism [17]. Few studies have demonstrated convincing associations between *ABCB1* polymorphisms and DILI development, though the *ABCB1* 3435T allele has been associated with reduced risk of nevirapine hepatotoxicity in several independent studies [6]-[8]. We, however, did not find this allele to have any major impact on the risk of DILI in the current study, possibly due to the lack of nevirapine cases in our ethnically different cohort.

In accordance with previous results the three *ABCB1* polymorphisms studied were found to be in linkage disequilibrium with low haplotype diversity. The two major haplotypes 1236C/2677G/3435C and 1236T/2677T/3435T were predicted to be present in approximately 80% of the cohort. The difference between *ABCB1* haplotypes in terms of transporter activity is unclear. Our study, however, does not support evidence for *ABCB1* haplotypes having differential effects on DILI development. Interestingly the *ABCB4* 1954A>G and *ABCB1* 3435T>C SNP pair were seen to have a high D' value, suggesting possible LD, though this is not supported by the low R^2^ value. Due to the low frequency of the *ABCB4* G allele and the large distance between the SNPs (>80 kbp) this finding should be considered with caution. Nevertheless, long distance LD has been reported in *ABCB1* stretching across to the nearby *ABCB4* gene [34].

Both MRP2 and P-gp have wide substrate specificity with certain degree of overlap, in addition to their co-localization on the hepatocyte canalicular membrane. We therefore hypothesised that reduced activity in one of these transporters may be compensated for by the other. Hence, specific allele combinations formed by these two genes could potentially shed light onto the underlying mechanism of DILI development. It is possible that putative *ABCB1*/*ABCC2* allele combinations effecting DILI development may require DILI cases with the same causative agent or those forming similar metabolites in terms of structural elements. Hence, the pooling of DILI cases induced by a wide range of causal agents could have diluted potentially different allele combination frequencies.

In conclusion, due to the complexity of the human efflux transporter system it is difficult to determine its potential role in DILI development. Our data do not support a role for the selected polymorphisms in ABC transporter genes *ABCB1*, *ABCB4* and *ABCC2* in DILI development in Spanish patients, neither individually nor in combinations. The -1774del allele in *ABCC2* was however restricted to DILI cases and could potentially contribute to enhanced DILI susceptibility. In addition, this study highlights the differential effect of specific ABC transporter alleles in ethnically different cohorts.
