# Correction: Selected *ABCB1*, *ABCB4* and *ABCC2* Polymorphisms Do Not Enhance the Risk of Drug-Induced Hepatotoxicity in a Spanish Cohort

**DOI:** 10.1371/journal.pone.0141400

**Published:** 2015-10-16

**Authors:** Eugenia Ulzurrun, Camilla Stephens, Francisco Ruiz-Cabello, Mercedes Robles-Diaz, Pablo Saenz-López, Hacibe Hallal, German Soriano, Eva Roman, M. Carmen Fernandez, M. Isabel Lucena, Raúl J. Andrade

There are errors in the Funding section. The correct funding information is as follows: This study was supported, in part, by research grants from the Agencia Española del Medicamento and Fondo de Investigación Sanitaria (PI12/00378). Fondo Europeo de Desarrollo Regional (FEDER). CIBERehd and Red Genómica del Cáncer are funded by Instituto de Salud Carlos III. The funders had no role in study design, data collection and analysis, decision to publish, or preparation of the manuscript.
